# WNT1, a target of miR-34a, promotes cervical squamous cell carcinoma proliferation and invasion by induction of an E-P cadherin switch via the WNT/β-catenin pathway

**DOI:** 10.1007/s13402-020-00506-8

**Published:** 2020-04-16

**Authors:** Baohua Li, Xuedong Guo, Na Li, Qin Chen, Junhua Shen, Xiaoxiu Huang, Genping Huang, Fenfen Wang

**Affiliations:** 1 Department of Gynecologic Oncology, Women’s Hospital, Zhejiang University School of Medicine, Hangzhou Zhejiang, People’s Republic of China; 2 Department of Pathology, Women’s Hospital, Zhejiang University School of Medicine, Hangzhou Zhejiang, People’s Republic of China

**Keywords:** Cervical squamous cell carcinoma, miR-34a, WNT1, E-cadherin, P-cadherin, Cadherin switch

## Abstract

**Purpose:**

Persistent infection with high-risk human papillomavirus (HR-HPV) is thought to play a prominent role in the initiation and progression of almost all cases of cervical cancer. Previously, we and others found that microRNA 34a (miR-34a) may be regulated by HR-HPV E6 to contribute to the development of cervical cancer. Here, we aimed to identify the oncogenic potential and clinical significance of a known miR-34a target, WNT1, in cervical squamous cell carcinoma (SCC) development and to investigate the associated mechanisms underlying cervical SCC cell proliferation and invasion.

**Methods:**

WNT1 and miR-34a expression levels were assessed in primary cervical lesions using immunohistochemistry and qRT-PCR, respectively. The cellular effects and the expression of its associated genes were examined in cervical SCC-derived Siha and Caski cells after siRNA-WNT1 (downregulation) or miR-34a mimic (upregulation) treatment. A cervical SCC xenograft mouse model was used to investigate the in vivo effects of miR-34a overexpression. HPV-16 E6/E7 expression was inhibited by gene promoter siRNA targeting, after which the levels of miR-34a and WNT1 were examined.

**Results:**

WNT1 protein upregulation was found to be associated with a poor prognosis in cervical SCC patients. In vitro assays in Siha and Caski cells revealed that WNT1 downregulation decreased cell proliferation and invasion, inhibited WNT/β-catenin activation and affected the expression of E-cadherin and P-cadherin. MiR-34a upregulation resulted in decreased WNT1 expression. An inverse correlation between miR-34a and WNT1 expression was also observed in primary cervical SCC tissues. In addition, we found that MiR-34a could regulate an E-cadherin to P-cadherin switch (E-P cadherin switch) to inhibit cell proliferation and tumorigenesis in vitro and in vivo via inactivation of the WNT1/β-catenin pathway. Finally, we found that decreased HPV-16 E6/E7 expression resulted in miR-34a upregulation and WNT1 downregulation in Siha and Caski cells.

**Conclusions:**

From our results we conclude that WNT1, as a target of miR-34a, can promote cervical SCC cell proliferation and invasion by induction of an E-P cadherin switch via the WNT1/β-catenin pathway. Our results may provide new options for the treatment of patients with cervical SCC.

**Electronic supplementary material:**

The online version of this article (10.1007/s13402-020-00506-8) contains supplementary material, which is available to authorized users.

## Introduction

Cervical cancer is one of the most common malignancies in females with over 527,000 new cases and 265,000 deaths worldwide per year [1, 2]. Persistent infection with high-risk human papillomavirus (HR-HPV) is regarded as a necessary factor in the initiation and progression of almost all cases of cervical cancer. HR-HPV E6/E7 can cause the degradation of p53 and retinoblastoma tumor suppressor family members to promote cell proliferation and invasion and to inhibit apoptosis [3]. Although it plays an important role in regulating the expressions of downstream genes, microRNAs (miRNAs) and signaling pathways, the exact mechanisms by which HR-HPV E6/E7 induces cervical cancer initiation and progression are not yet fully elucidated. Our and other studies showed that miR-34a, as an important tumor-suppressive miRNA, may be regulated by HR-HPV E6 to contribute to these processes in a p53-dependent manner [4, 5].

The cadherin superfamily is an ancient family of adhesion molecules of which the classical cadherins are most extensively studied. Deregulation and abnormal expression of classical cadherins have not only been detected in different types of cancer, but have also been found to contribute to their initiation and development [6]. In our previous study, aberrantly expressed classical cadherins, including epithelial cadherin (E-cadherin) and placental cadherin (P-cadherin), were detected in cervical lesions and to be significantly associated with a poor prognosis and survival in early-stage cervical squamous cell carcinoma (SCC) patients [7]. In addition, we found that exogenous expression of HPV-16 E6/E7 in cervical cell lines significantly increased P-cadherin levels and decreased E-cadherin levels, consequently leading to an E-cadherin to P-cadherin switch (E-P cadherin switch) [8]. These results indicate that HPV-16 E6/E7 may be involved in cervical cancer initiation and progression by regulating the E-P cadherin switch. However, the exact role of HPV-16 E6/E7 in the E-P cadherin switch in SCC has so far remained unexplored.

The human Wingless-type (WNT) genes constitute an evolutionarily conserved family of 19 members encoding secreted glycoproteins that may regulate the β-catenin-dependent WNT signaling pathway. Aberrant WNT/β-catenin pathway activation has been reported to promote tumor growth, metastasis and drug resistance [9]. More importantly, targeting the WNT/β-catenin pathway is regarded as a valuable therapeutic option in many malignancies [10]. Recently, a genome-wide expression study in cervical SCC samples with HPV-16 infection revealed that activation of the WNT/β-catenin pathway was regulated by HPV-16 [11]. Therefore, it was hypothesized that activation of the WNT/β-catenin pathway represents another hit in the multistep process of cervical carcinogenesis in addition to persistent HR-HPV infection [12]. An association between the WNT/β-catenin pathway and cadherin switching has been well-studied. In non-small cell lung cancer, for example, it has been found that FOXP3 activates the WNT/β-catenin pathway, induces a cadherin switch and, by doing so, contributes to tumor growth and metastasis [13]. It has also been found that MgCl_2_ and ZnCl_2_ treatment can induce cytoskeleton remodeling in human umbilical vein endothelial cells and stimulate a cadherin switch via activation of the WNT/β-catenin pathway, characterized by a decrease in E-cadherin and an increase in N-cadherin [14]. WNT family member 1 (WNT1) has been identified as a target of miRNA-34a (miR-34a) [15], and it has been found that miR-34a can affect colon cancer progression [16] and inhibit breast cancer proliferation [17] via inactivation of the WNT/β-catenin pathway. We, therefore, set out to evaluate whether miR-34a may target the WNT1/β-catenin pathway and, subsequently, induce an E-P cadherin switch during cervical cancer development regulated by HPV-16 E6/E7.

In the present study, we examined the expression of WNT1 in normal cervical epithelium and cervical SCC tissues, and explored the effect of decreased WNT1 expression on cervical SCC cell behavior and the expressions of E-cadherin and P-cadherin, as well as the expression of molecules associated with the WNT/β-catenin pathway in HPV-16 positive cervical SCC-derived cell lines. Furthermore, we assessed the level of miR-34a in the same cervical SCC tissues in which WNT1 expression was detected. To further explore how miR-34a contributes to cervical SCC cell proliferation, invasion and tumorigenesis by the regulation of WNT1 expression, both in vitro and in vivo assays were used. Finally, associations between HPV-16 E6/E7 and miR-34a and WNT1 expression levels were investigated.

## Materials and methods

### Patients and tissue specimens

A total of 131 patients with cervical squamous cell carcinoma (SCC) was enrolled in the present study. All patients underwent radical hysterectomy with pelvic lymph node dissection and did not receive any anticancer therapy prior to their surgery in Women’s Hospital, Zhejiang University School of Medicine, between January 2007 and December 2009. Additionally, control normal cervical epithelial tissues were collected from patients (*n* = 50) who underwent hysterectomy due to benign gynecologic diseases. After surgical removal, all cervical tissues were immediately snap frozen in liquid nitrogen and stored at −70 °C until use. The HR-HPV infection status was determined using a hybridization capture 2 (HC2). Hybribio Rapid HPV GenoArray test kit (Hybribio Limited, Hong Kong, CHN) allowing the assessment of different HR-HPV types in HR-HPV infected cervical SCC tissues, including 15 high-risk types (16, 18, 31, 33, 35, 39, 45, 51, 52, 53, 56, 58, 59, 66 and 68). The use of all tissue specimens was approved by the Hospital Research Ethics Committee. Informed consent was obtained from all patients.

### Cell culture and transfection

The HPV-16 positive cervical cancer cell lines Siha (Catalogue number: TCHu113) and Caski (Catalogue number: TCHu137) and the HPV negative cervical cancer cell line C33A (Catalogue number: TCHu176) were purchased from the Shanghai Chinese Academy of Science cell bank (Shanghai, CHN). A WNT1 targeting small interfering RNA (siRNA) (siR-WNT1), a HPV-16 E6/E7 promoter-targeting siRNA and a siRNA negative control (siR-Cont) were chemically synthesized by GeneChem Co., Ltd. (Shanghai, CHN). The siRNA sequences are shown in Supplementary Table 1. A miRNA-34a mimic (miR-34a) and a miRNA mimic negative control (miR-Cont) were chemically synthesized by Biomics Biotechnology Inc. (Jiangsu, CHN). The siRNAs and miRNAs were transfected separately into Siha and Caski cells at final concentrations of 30 nmol/L (siRNA) or 50 nmol/L (miRNA), according to the manufacturer’s instructions. For the in vivo animal experiments agomiR-miR-34a and agomiR scramble were purchased from GenePharma Co., Ltd. (Shanghai, CHN).

### Lithium chloride (LiCl) treatment

LiCl was purchased from Sigma Company (St. Louis, MO, USA) and dissolved in phosphate buffer solution (PBS). Siha or Caski cells were cultured overnight and treated with LiCl at a final concentration of 30 mmol/L for further study.

### Cell counting kit-8 (CCK-8) assay

Cells were cultured separately in 96-well plates overnight and subsequently transfected with siR-WNT1 or miR-34a mimics. At the end of the indicated time points (24, 48, 72 and 96 h post-transfection) the cells were incubated with 5 mg/ml CCK-8 solution (Dojindo, Kumamoto, JPN) for 4 h. Next, the absorbance of the samples was recorded at 450 nm using a GENios multifunction reader. The data presented are based on three independent experiments.

### Cell cycle and apoptosis assays

Flow cytometry was used for cell cycle and apoptosis analyses at 48 h post-transfection. For the cell cycle analyses, the cells were collected by trypsinization and fixed with 75% ethanol at −20 °C for 24 h. Next, the cells were incubated with a propidium iodide/PBS staining buffer (KeyGen, Nanjing, CHN) at 37 °C for 30 min, after which the percentages of G1, S and G2/M phase cells were calculated using Cell Quest software. For the apoptosis assays, the cells were treated by Annexin-V and propidium iodide (KeyGen, Nanjing, CHN) and analyzed within 30 min after staining. Quantification of fluorescence was performed by flow cytometry.

### Migration and invasion assays

The cells were suspended in 200 ul serum-free medium and seeded into the upper chamber of a Transwell insert (Corning, NY, USA). The lower chamber was filled with optimal medium supplemented with 10% fetal bovine serum. After 24 h, the cells on the upper surface of the membrane were wiped off, while the cells that migrated to the lower surface were fixed, stained with crystal violet and counted in four randomly selected fields using an inverted microscope. To assess the invasive capacity of the cells the same protocol was used, except that the inserts were precoated with Matrigel (Corning, NY, USA). The data are presented based on three independent experiments.

### Tumor formation in BALB/c-nu mice

BALB/c-nu mice (female, 4–6 weeks old and 16–20 g) were purchased from the Laboratory Animals center of Zhejiang University. All animal experiments were carried out in accordance with the Guide for the Care and Use of Laboratory Animals of Zhejiang University. After subcutaneous inoculation of Siha cells to establish xenograft models, the mice were randomly divided into two groups (*n* = 6/group). One group was injected with 5 nM agomiR-miR-34a, while the other group was injected with 5 nM agomiR scramble through the lateral tail vein every 4 days for 7 weeks. From the first day after injection, the tumor volumes were measured every 3 days using a caliper. On day 54 the mice were sacrificed, after which the tumors were removed, photographed and weighed. The tumor tissues were recovered and subsequently processed for TUNEL (KeyGen, Nanjing, CHN) and Western blotting assays.

### RNA extraction and quantitative RT-PCR (qRT-PCR)

Total RNA was isolated from both cervical tissues and transfected cells using the TRIzol method, according to the manufacturer’s instructions. Subsequent qRT-PCR for mRNA and miRNA quantifications were performed as described before. For mRNA quantification, glyceraldehyde-3-phosphate dehydrogenase (GAPDH) was used as internal reference. For miRNA quantification, small nucleolar RNA RNU6 (U6) was used as endogenous control. The primers used are listed in Supplementary Table 1. The miR-34a and U6 primers were synthesized and provided by Biomics Biotechnology Inc. (Jiangsu, CHN).

### Western blotting

For this purpose, cells and tissues were collected in ice-cold PBS and lysed in RIPA buffer supplemented with a proteinase inhibitor cocktail. Next, Western blotting was performed as described before. The primary antibodies used are listed in Supplementary Table 2.

### Immunohistochemical staining

WNT1 protein expression was detected using immunohistochemical staining in 181 primary cervical tissues. Briefly, four-micron-thick sections were obtained from paraffin-embedded tissues and, subsequently, incubated with a primary antibody directed against WNT1 for 1 h at room temperature. The immunohistochemical staining of each sample was scored depending on staining intensity and the percentage of cells stained. Specifically, the staining intensity was assigned a rating from 0 to 3: 0 means negative; 1 means weakly positive; 2 means moderately positive; 3 means strongly positive. The staining distribution was scored as 0 for < 10%; 1 for 10 to < 25%; 2 for 25 to < 50%; 3 for 50 to < 75%; and 4 for 75 to < 100%. Finally, the intensity and percentage scores were multiplied to get an overall score. Scores < 4 were considered as low and ≥ 4 as high expression.

### Follow-up assays

The 131 patients with primary cervical SCC were followed-up postoperatively through oral interviews at the clinic or by phone calls. Regional tumor recurrence, distant metastasis and patient survival were recorded. Disease-free survival (DFS) and overall survival (OS) times were calculated from the day of the surgery until the day of recurrence or death. The last day of follow-up was December 31, 2016. The mean follow-up period was 90.88 months (range 12–118 months) at the time of study closure. The recurrence and death rates were recorded during the follow-up period. We recorded 22 recurrences (22/131, 16.79%) and 20 deaths (20/131, 15.27%).

### Statistical analysis

All statistical analyses were performed using SPSS 23.0 for windows software. The data are presented as the mean ± standard deviation (SD). For both in vitro and in vivo assays, a Student’s t test was used to determine statistical differences between two groups. One-way ANOVA was used to determine statistical differences between multiple testing groups. The Pearson’s chi-square test was used to analyze relationships between WNT1 or miR-34a levels and clinicopathological characteristics, as well as between WNT1 and miR-34a levels in 131 SCC tissues. Survival curves were plotted using the Kaplan-Meier method and compared by log-rank test. In the univariate and multivariate analyses, the Cox proportional hazard method was used to identify independent predictors of survival. All statistical tests were two-sided, and *p* values < 0.05 were considered to be statistically significant (* means *p* < 0.05; ** means *p* < 0.01).

## Results

### WNT1 protein expression is up-regulated in cervical SCC tissues and correlates with poor prognostic parameters

**Fig. 1:**
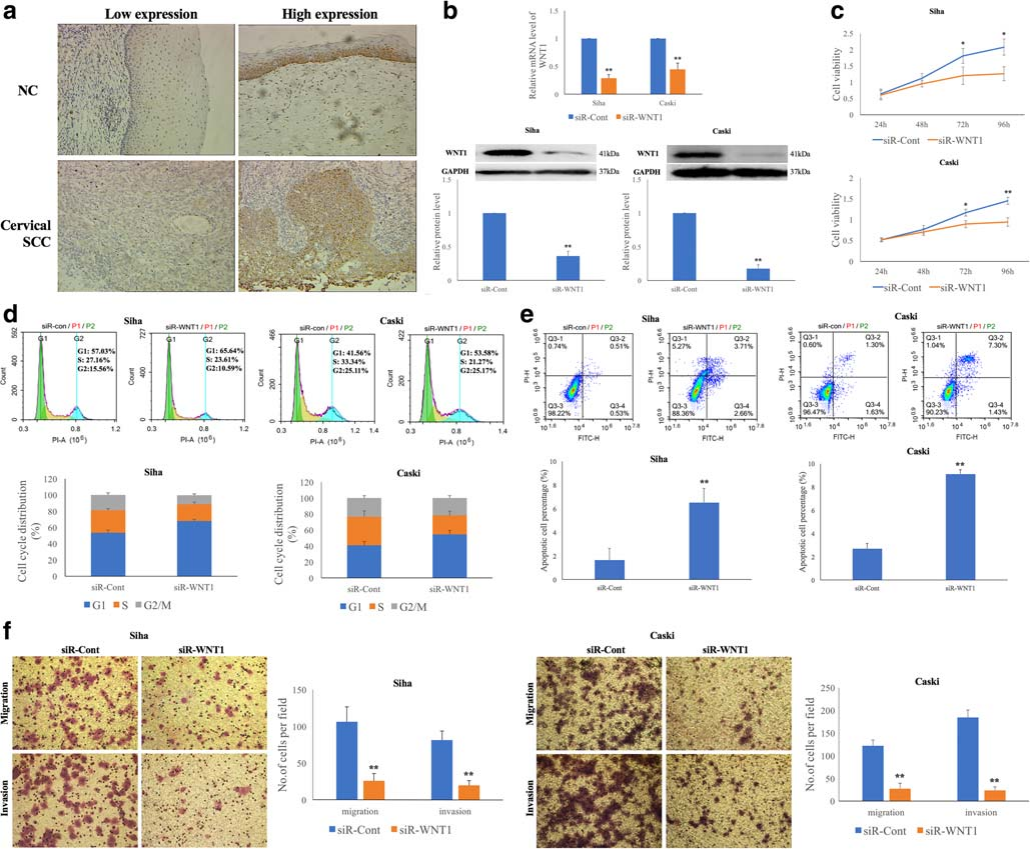
**SiRNA-mediated WNT1 silencing suppresses the proliferation and invasion of HPV-16 positive cervical SCC cells.a** Strong membranous and cytoplasmic staining of WNT1 in cervical tissues (magnification ×200). **b** WNT1 silencing effectively down-regulates WNT1 mRNA and protein levels 48 h post-transfection in Siha and Caski cells. WNT1 silencing results in significant inhibition of cervical SCC cell proliferation (**c**), accumulation of cervical SCC cells in the G1 phase of the cell cycle (**d**), promotion of cervical SCC cell apoptosis (**e**) and suppression of cervical SCC cell migration and invasion (**f**)

To evaluate the expression of WNT1 protein in cervical specimens and to further investigate correlations of WNT1 expression with clinical parameters, we collected 50 normal cervical epithelium (NC) and 131 primary cervical SCC tissues. We found that WNT1 immunoreactivity was predominantly localized in the cellular membrane and cytoplasm, but was less evident in the nucleus (Fig. 1a). An increased WNT1 expression was observed in 69 of the 131 cervical SCC tissues compared to 2 of the 50 NC tissues, and this difference was statistically significant (*p* = 2.01E^−9^) (Supplementary Table 3). We also determined correlations of WNT1 expression with clinical parameters in the cervical SCC tissues (Table 1). We found that increased WNT1 expression was significantly associated with different HR-HPV types (*p* = 0.035), FIGO stage (*p* = 0.042), depth of stromal invasion (*p* = 0.003), lymph vascular space invasion (LVSI) (*p* = 0.020) and lymph node metastasis (LNM) (*p* = 0.011).

**Table 1:** Association between WNT1 expression or miR-34a level and clinical parameters in 131 patients with cervical squamous cell carcinoma (SCC)

**Characteristic**	n	WNT1, n (%)		*p* value	miR-34a, n (%)		*p* value
		Low	High		Low	High	
**Patient age (year)**				0.958			0.243
≤ 40	23	11 (8.4)	12 (9.2)		15 (11.5)	8 (6.1)	
> 40	108	51 (38.9)	57 (43.5)		83 (63.3)	25 (19.1)	
**Status of HR-HPV**				0.570			0.317
Positive	94	45 (44.1)	49 (48.0)		68 (66.7)	26 (25.5)	
Negative	8	3 (2.9)	5 (4.9)		7 (6.9)	1 (1.0)	
**Different HR-HPV types**				***0.035***			***0.001***
HPV-16/18 types	32	10 (12.7)	22 (27.8)		29 (36.7)	3 (3.8)	
Others HR-HPV types	47	26 (32.9)	21 (26.6)		28 (35.4)	19 (24.1)	
**FIGO stage**				***0.042***			***0.036***
I	97	51 (38.9)	46 (35.1)		68 (51.9)	29 (22.1)	
II	34	11 (8.4)	23 (17.6)		30 (22.9)	4 (3.1)	
**Differentiation**				0.457			0.770
Well/moderate	109	50 (38.2)	59 (45.0)		81 (61.8)	28(21.4)	
Poor	22	12 (9.2)	10 (7.6)		17 (13.0)	5 (3.8)	
**Tumor size (cm)**				0.819			0.366
< 4	115	54 (41.2)	61 (46.6)		88 (67.2)	27 (20.6)	
≥ 4	16	8 (6.1)	8 (6.1)		10 (7.6)	6 (4.6)	
**Stromal invasion**				***0.003***			***0.001***
< 2/3	82	47 (35.9)	35 (26.7)		53 (40.5)	29 (22.1)	
≥ 2/3	49	15 (11.4)	34 (26.0)		45 (34.4)	4 (3.1)	
**Vaginal wall extension**				0.981			1.0
Yes	17	8 (6.1)	9 (6.9)		13 (9.9)	4 (3.1)	
No	114	54 (41.2)	60 (45.8)		85 (64.9)	29 (22.1)	
**Parametrial extension**				0.447			0.099
Yes	11	4 (3.1)	7 (5.3)		11 (8.4)	0 (0.0)	
No	120	58 (44.3)	62 (47.3)		87 (66.4)	33 (25.2)	
**Endometrial extension**				0.619			0.731
Yes	3	1 (0.8)	2 (1.5)		3 (2.3)	0 (0.0)	
No	128	61 (46.6)	67 (51.1)		95 (72.5)	33 (25.2)	
**LVSI**				***0.020***			***0.007***
Yes	54	19 (14.5)	35 (26.7)		47 (35.9)	7 (5.3)	
No	77	43 (32.8)	34 (26.0)		51 (38.9)	26 (19.8)	
**Surgical margin**				0.057			0.814
Clear	124	61 (46.6)	63 (48.1)		92 (70.2)	32 (24.4)	
Involved	7	1 (0.8)	6 (4.5)		6 (4.6)	1 (0.8)	
**LNM**				***0.011***			***0.014***
Yes	22	5 (3.8)	17 (13.0)		21(16.0)	1 (0.8)	
No	109	57 (43.5)	52 (39.7)		77 (58.8)	32 (24.4)	

FIGO, International Federation of Gynecology and Obstetrics; LVSI, lymph vascular space invasion; LNM, lymph node metastasis; HR-HPV, high-risk human papillomavirus. Of 131 SCC patients, no information about HR-HPV infection status was available in 29 cases; Among 94 HR-HPV infected cases, HPV-16/18 types: 32 cases, other HR-HPV types: 47 cases, unidentified types: 15 cases

### WNT1 downregulation results in decreased proliferation and invasion of HPV-16 positive cervical SCC cells

To investigate the role of WNT1 in cervical cancer development, we first assessed the level of WNT1 expression in HPV-16 positive Siha and Caski cells and HPV-negative C33A cells. Using Western blotting, we found that the WNT1 expression level was remarkably higher in the HPV-16 positive than in the HPV-negative cervical SCC cells (Supplementary Fig. 1a). Therefore, we selected the Siha and Caski cells for our further analyses. We found that siRNA-mediated WNT1 targeting in Siha and Caski cells effectively downregulated the mRNA and protein levels of WNT1 at 48 h post-transfection (Fig. 1b). Subsequently, we examined the proliferation rates of Siha and Caski cells using CCK-8 analyses, and found that WNT1 expression inhibition resulted in a reduced cellular growth at 72 and 96 h post-transfection (*p* < 0.05) (Fig. 1c). We also tested cell cycle progression patterns using PI staining and found that WNT1 expression inhibition in Siha and Caski cells led to a significant accumulation of cells in the G1 phase and a reduction of cells in the S and G2/M phases (*p* < 0.05) (Fig. 1d). Additionally, we found that the apoptosis rate was increased in WNT1 downregulated cells as determined by using an Annexin V&PI apoptosis detection kit, compared to the respective negative controls (*p* < 0.01) (Fig. 1e). These data suggest that decreased WNT1 expression may inhibit cervical SCC cell growth through influencing cell cycle progression and apoptosis rates. Finally, our data indicate that WNT1 downregulation results in decreased cervical SCC cell migration (*p* = 0.003 and *p* = 0.001) and invasion (*p* = 0.002 and *p* = 1.12E^−4^) in Siha and Caski cells, respectively (Fig. 1f).

### WNT1 downregulation affects E-cadherin and P-cadherin expression via the WNT/β-catenin pathway in HPV-16 positive cervical SCC cells

**Fig. 2:**
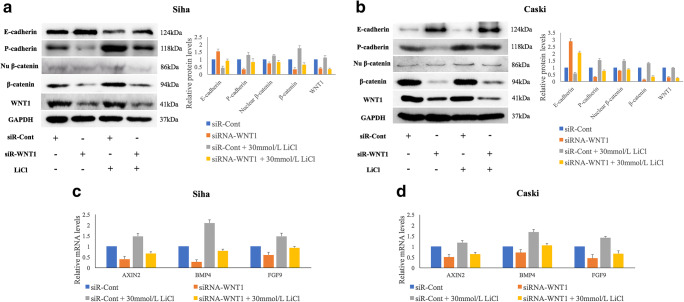
**SiRNA-mediated WNT1 silencing significantly affects E-cadherin and P-cadherin expression and reduces the expression of associated factors of the WNT/β-catenin pathway in HPV-16 positive cervical SCC cells.** WNT1 silencing significantly affects the expression of E-cadherin and P-cadherin, and suppresses the expression of total and nuclear β-catenin 48 h post-transfection in Siha (**a**) and Caski (**b**) cells as determined by Western blotting. WNT1 silencing also results in reduced AXIN2, BMP4 and FGF9 expression as determined by qRT-PCR in Siha (**c**) and Caski (**d**) cells. LiCl treatment partially recovers the expression of these genes in WNT1 silenced Siha and Caski cells (**a-d**). Data are presented as mean ± SD; *n* = 3

To reveal a mechanistic link between WNT1 expression and cell growth and invasiveness, we examined the levels of classical cadherins, including E-cadherin, P-cadherin and N-cadherin (neural), in Siha and Caski cells with and without siR-WNT1 transfection. We found that decreased WNT1 expression resulted in upregulation of E-cadherin expression (*p* = 0.005, *p* = 0.001) and downregulation of P-cadherin expression (*p* = 0.002, *p* = 1.09E^−4^) at 48 h post-transfection (Fig. 2a, b), respectively. The protein level of N-cadherin was found to be too low to be detected in the respective cells (Supplementary Fig. 1c). Furthermore, we examined the protein levels of total β-catenin and nuclear β-catenin, as well as the mRNA levels of three downstream target genes (AXIN2, BMP4 and FGF9) of the WNT/β-catenin pathway (Fig. 2, a-d). As expected, siR-WNT1 transfection resulted in a significant suppression of the expressions levels of total β-catenin (*p* = 0.001, *p* = 0.003) and nuclear β-catenin (*p* = 0.041, *p* = 0.003) in Siha and Caski cells. Additional qRT-PCR analyses revealed that WNT1 downregulation resulted in reduced AXIN2 (*p* = 1.39E^−4^, *p* = 0.020), BMP4 (*p* = 2.55E^−4^, *p* = 0.020) and FGF9 (*p* = 0.030, *p* = 0.030) expression levels. Lithium chloride (LiCl), which has been shown to stimulate WNT/β-catenin pathway activity and to increase the expression of total β-catenin and nuclear β-catenin, was added to the Siha and Caski cells at a final concentration 30 mmol/L with or without siR-WNT1 transfection. We found that LiCl administration attenuated the inhibition of total and nuclear β-catenin protein expression and the suppression of AXIN2, BMP4 and FGF9 expression induced by siR-WNT1 transfection and, subsequently, partially recovered the expression of E-cadherin and P-cadherin in siR-WNT1 transfected Siha and Caski cells (Fig. 2, a-d).

### MiR-34a alters E-cadherin and P-cadherin levels via the WNT1/β-catenin pathway in HPV-16 positive cervical SCC cells

**Fig. 3:**
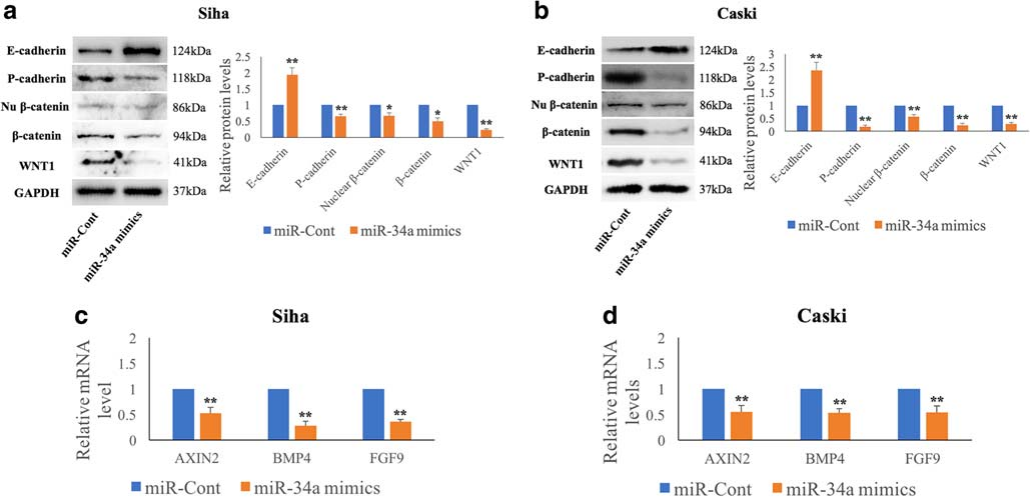
**MiR-34a targets the WNT1/β-catenin pathway and, subsequently, regulates the E-P cadherin switch.** Exogenous miR-34a over-expression, using miR-34a mimics, significantly suppresses the expression of WNT1 and total and nuclear β-catenin protein, and affects the expression of E-cadherin and P-cadherin at 48 h post-transfection in HPV-16 positive cervical SCC Siha (**a**) and Caski (**b**) cells as determined by Western blotting. Exogenous miR-34a over-expression also results in reduced AXIN2, BMP4 and FGF9 expression in Siha (**c**) and Caski (**d**) cells as determined by qRT-PCR. Data are presented as mean ± SD; n = 3

WNT1 has been reported to be a direct target of miR-34a in several types of cancer. Using qRT-PCR, we found that the miR-34a level was significantly lower in Siha and Caski cells than in C33A cells (Supplementary Fig. 1b). Next, we used a miR-34a mimic to upregulate the miR-34 level in Siha and Caski cells and, subsequently, assessed their WNT1 expression. We found that increased miR-34a expression resulted in reduced WNT1 mRNA and protein levels at 48 h post-transfection (Supplementary Fig. 1d, e). Additionally, we used qRT-PCR to assess the miR-34a level in the same cervical SCC tissues in which expression of the WNT1 protein was detected (see above). A significant inverse correlation (*p* = 0.01) between miR-34a and WNT1 expression was noted (Supplementary Table 4). Taken together, our data suggest that WNT1 is negatively regulated by miR-34a at the transcriptional level in cervical SCC cells. Subsequently, we assessed total and nuclear β-catenin expression levels using Western blotting, and AXIN2, BMP4 and FGF9 mRNA levels using qRT-PCR after transfection of Siha and Caski cells with miR-34a mimics. We observed significantly decreased total β-catenin (*p* = 0.012, *p* = 0.003) and nuclear β-catenin (*p* = 0.029, *p* = 0.001) protein levels, and decreased AXIN2 (*p* = 0.002, *p* = 0.004), BMP4 (*p* = 1.31E^−4^, *p* = 0.001) and FGF9 (*p* = 1.1E^−5^, *p* = 0.004) mRNA levels in miR-34a-overexpressing Siha and Caski cells, respectively (Fig. 3, a-d). Our Western blotting results additionally revealed that exogenous miR-34a expression significantly upregulated the E-cadherin (*p* = 0.002, *p* = 0.001) and downregulated the P-cadherin (*p* = 0.008, *p* = 0.002) levels, respectively. We failed to detect N-cadherin expression in these cells using Western blotting (Supplementary Fig. 1c).

### Exogenous miR-34a overexpression reduces the proliferation and invasion of HPV-16 positive cervical SCC cells

**Fig. 4:**
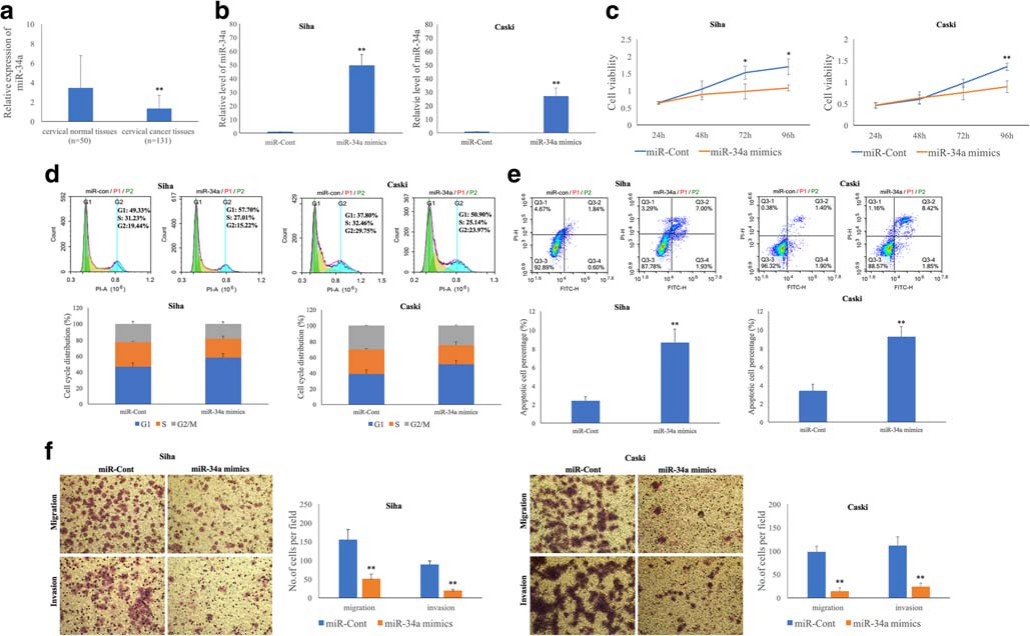
**Exogenous miR-34a over-expression reduces the proliferation and invasion of HPV-16 positive cervical SCC cells.a** The miR-34a level is lower in cervical SCC tissues (*n* = 131) compared to that in normal cervical tissues (*n* = 50) as determined by qRT-PCR. **b** Exogenous miR-34a over-expression significantly increases the miR-34a level in Siha and Caski cells as determined by qRT-PCR. Exogenous miR-34a over-expression inhibits cervical SCC cell proliferation (**c**), blocks cervical SCC cell cycle progression (**d**), increases cervical SCC cell apoptosis (**e**) and decreases cervical SCC cell migration and invasion (**f**)

To verify the role of miR-34a in cervical cancer initiation and progression, we upregulated the miR-34a level in Siha and Caski cells using miR-34a mimics (Fig. 4b). We found that the proliferation rates of Siha cells (*p* = 0.030, *p* = 0.012) at 72 and 96 h and Caski cells (*p* = 0.008) at 96 h post-transfection were significantly decreased (Fig. 4c), that the apoptosis rates were increased and that cell cycle progression was blocked at the G1 phase at 48 h post-transfection, compared to the respective negative controls (*p* < 0.05) (Fig. 4d, e). Moreover, we found using Transwell migration and invasion assays that the migration and invasion capacities of Siha (*p* = 0.003 and *p* = 0.001) and Caski (*p* = 0.001 and *p* = 0.002) cells transfected with miR-34a mimics were significantly reduced (Fig. 4f).

### MiR-34a suppresses tumor growth and promotes apoptosis via inactivation of the WNT1/ β-catenin pathway in vivo

**Fig. 5:**
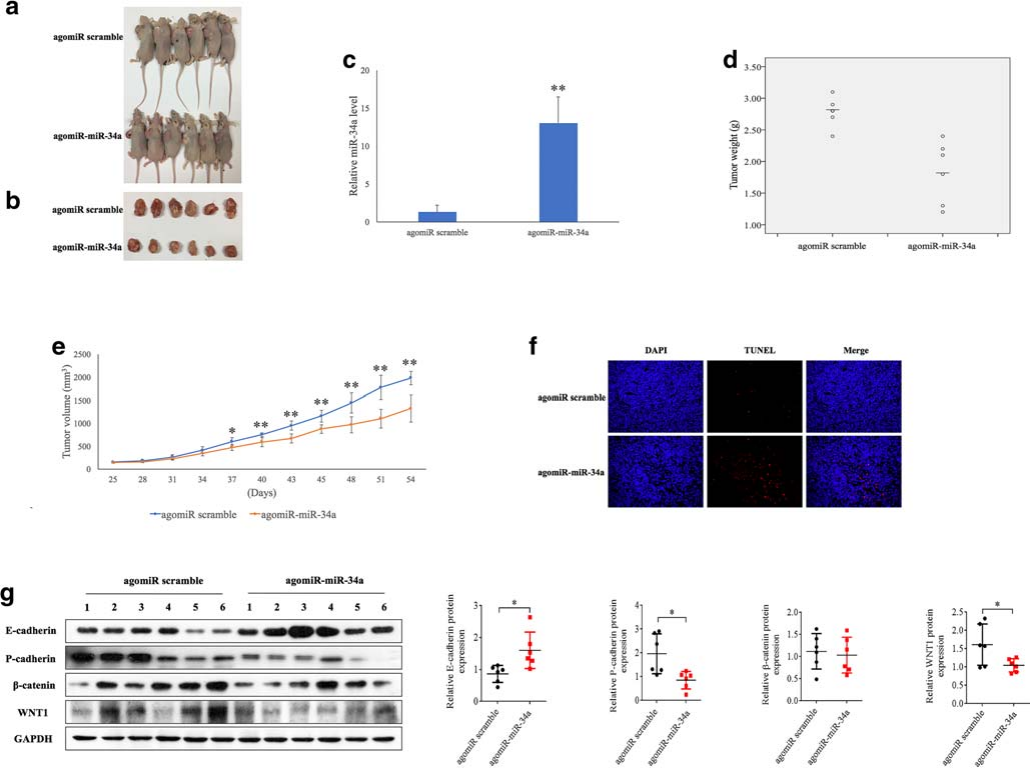
**MiR-34a suppresses in vivo tumor growth in BALB/c-nu mice via inactivation of the WNT1/β-catenin pathway.a, b** Xenograft tumors after 5 nM agomiR-miR-34a or agomiR scramble treatment. **c** qRT-PCR results showing that the miR-34a levels are significantly higher in the agomiR-miR-34a group than in agomiR scramble group. Tumor weights (**d**) and volumes (**e**) measured in the agomiR-miR-34a and agomiR scramble groups. **f** TUNEL assay confirming the effect of miR-34a overexpression on the apoptotic rates in the tumor tissues. **g** Western blotting showing aberrant WNT1, β-catenin, E-cadherin and P-cadherin expression in tumor tissues from the two treatment groups

To assess the tumor suppressive role of miR-34a overexpression in vivo, we constructed a BALB/c-nu mouse xenograft model by injecting Siha cells. We found that the mice that were subsequently injected with agomiR-miR-34a showed a slower tumor growth than those injected with agomiR scramble (*p* < 0.05) (Fig. 5, a-e). Consistently, we found that the tumor weights in the agomiR-miR-34a group were significantly lower than those in the agomiR scramble group (*p* = 0.001). To determine the apoptotic rates in the tumor tissues between different treatment groups a TUNEL assay was used. Higher apoptotic rates in tumor tissues extracted from the agomiR-miR-34a group than in those from the agomiR scramble group were noted following 7 injections (Fig. 5f). Consistent with these results, we found that the WNT1 and P-cadherin protein levels were significantly decreased, and that the E-cadherin protein level was significantly increased in the agomiR-miR-34a group, whereas no difference in β-catenin protein levels were observed between the two groups, as measured by Western blotting (Fig. 5g). Collectively, these data indicate that miR-34a may regulate the E-P cadherin switch to inhibit in vivo tumor growth in a mouse xenograft model, at least in part, by targeting the WNT1/β-catenin pathway.

### MiR-34a expression is frequently decreased in SCC tissues and is associated with poor prognostic clinical parameters

In contrast to the WNT1 protein expression level, we found that the miR-34a level in the same SCC tissues was significantly decreased (*p* = 4.7E^−5^) compared to that of normal cervical tissues (Fig. 4a). Furthermore, we assessed correlations between miR-34a expression levels and clinical parameters in cervical SCC tissues (Table 1). Based on our qRT-PCR results, a 75th percentile of 1.32 was used as cut-off point for patients with a high or low miR-34a level, respectively. Among 131 SCC tissues tested, 33 exhibited high miR-34a levels and 98 low miR-34a levels. We found that low miR-34a levels were significantly associated with different HR-HPV types (*p* = 0.001), FIGO stage (*p* = 0.036), depth of stromal invasion (*p* = 0.001), LVSI (*p* = 0.007) and LNM (*p* = 0.014).

### HPV-16 E6/E7 simultaneously regulates miR-34a and WNT1 expression in HPV-16 positive cervical SCC cells

**Fig. 6:**
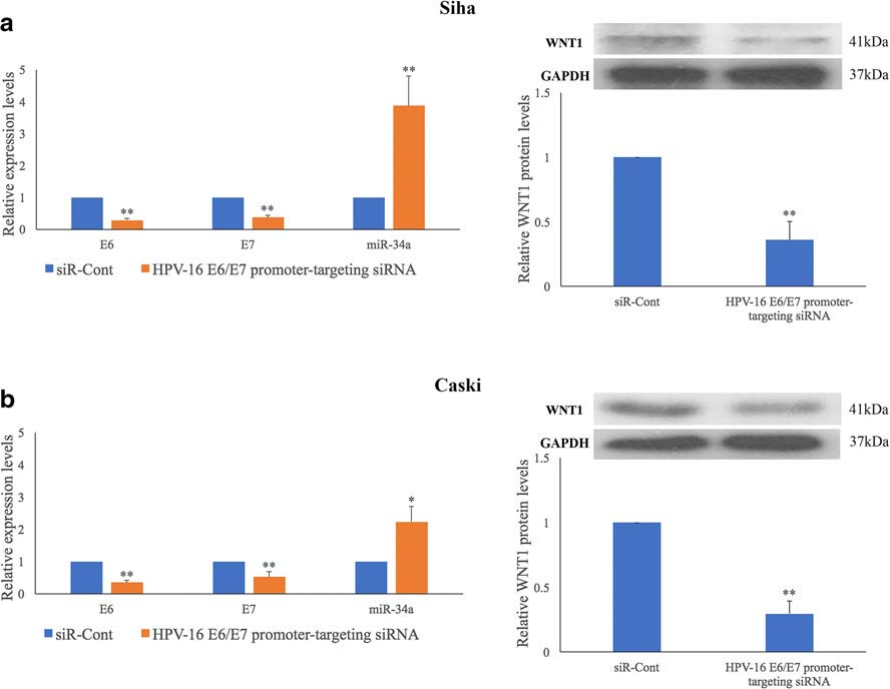
**HPV-16 E6/E7 regulates miR-34a and WNT1 expression in HPV-16 positive cervical SCC cells.** Promoter targeting siRNA-mediated silencing of HPV-16 E6/E7 expression results in up-regulation of miR-34a and down-regulation of WNT1 expression in Siha (**a**) and Caski (**b**) cells

Previously, we found that HPV-16 E6/E7 may regulate an E-P cadherin switch to promote cervical SCC cell growth and invasion [8]. To determine the effect of HPV-16 E6/E7 on the expression of miR-34a and WNT1, we reduced the level of E6 and E7 in HPV-16 positive Siha and Caski cells using a HPV-16 E6/E7 promoter-targeting siRNA [18]. We found that the reduction in HPV-16 E6/E7 expression resulted in miR-34a upregulation (*p* = 0.006 and *p* = 0.047), as determined by qRT-PCR, and downregulation of WNT1 expression (*p* = 0.002 and *p* = 0.007), as determined by Western blotting, in Siha and Caski cells (Fig. 6a, b).

### WNT1 and miR-34a expression levels are associated with patient survival

**Fig. 7:**
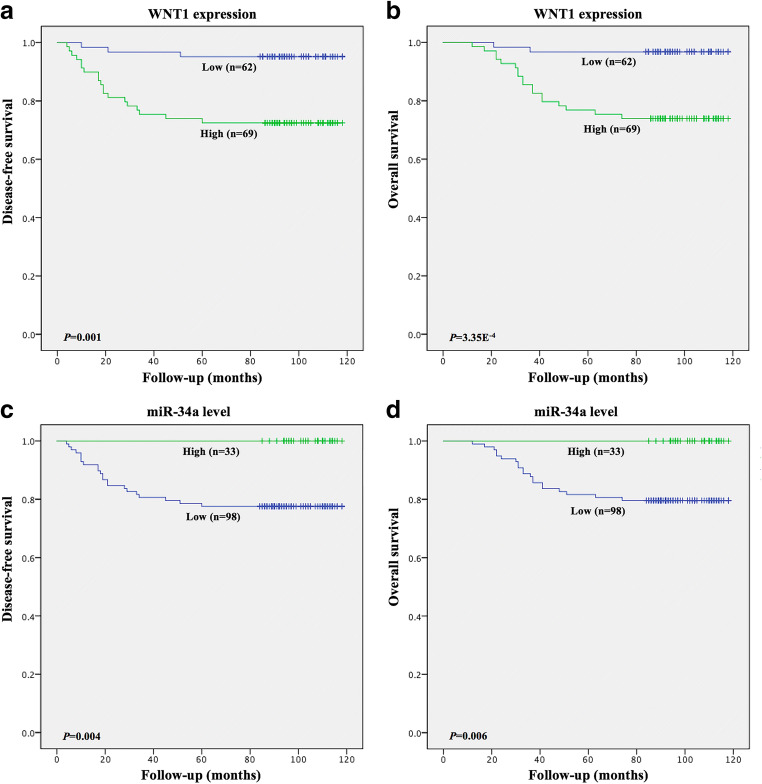
**High WNT1 and low miR-34a expression are significantly associated with a shorter disease-free survival (DFS) and overall survival (OS) time in SCC patients.** Kaplan-Meier curves showing associations between aberrant WNT1 and miR-34a expression with DFS (**a, c**) and (OS) (**b, d**) in 131 patients with early-stage cervical SCC

To investigate the prognostic value of WNT1 and miR-34a expression, survival curves were used to determine their association with disease-free survival (DFS) and overall survival (OS) in 131 cervical SCC patients. Significance was assessed using univariate and multivariate Cox regression models. Kaplan-Meier analysis revealed that high WNT1 expression and low miR-34a expression were associated with shorter DFS (*p* = 0.001 and *p* = 0.004) and OS (*p* = 3.35E^−4^ and *p* = 0.006) times (Fig. 7 and Table 2). More importantly, we found using univariate and multivariate analyses that elevated WNT1 expression, besides FIGO stage and LNM, served as an independent prognostic factor of DFS (*p* = 0.047) in cervical SCC patients. These data suggest that WNT1, as a target of miR-34a, can predict the prognosis and survival of cervical SCC patients.

**Table 2:** Univariate and multivariate analyses of associations between prognostic value and disease-free and overall survival rates in 131 patients with cervical squamous cell cancer (SCC)

Characteristic	Disease-free survival			Overall survival		
	HR	95% CI	*p value*	HR	95% CI	*p value*
Univariate analyses						
Age	0.701	0.259–1.901	0.485	1.145	0.336–3.908	0.829
Status of HR-HPV	1.312	0.173–9.937	0.792	1.203	0.158–9.152	0.858
Different HR-HPV types	1.307	0.681–2.511	0.421	1.279	0.648–2.524	0.478
FIGO stage	7.866	3.200–19.334	7.1E^−6^	8.420	3.230–21.954	1.3E^−5^
Differentiation	0.801	0.237–2.708	0.722	0.872	0.256–2.975	0.827
Tumor size	1.754	0.593–5.184	0.309	1.382	0.405–4.719	0.605
Stromal invasion	13.256	3.918–44.854	3.2E^−5^	11.708	3.427–40.003	8.7E^−5^
Vaginal wall extension	2.159	0.796–5.855	0.131	2.434	0.884–6.700	0.085
Parametrial extension	6.409	2.491–16.490	1.2E^−4^	6.892	2.632–18.041	8.4E^−5^
Endometrial extension	4.597	1.073–19.696	0.040	2.332	0.312–17.441	0.409
LVSI	5.723	2.110–15.523	0.001	5.000	1.816–13.766	0.002
Surgical margin	5.243	1.771–15.520	0.003	3.620	1.059–12.368	0.040
LNM	17.188	6.947–42.526	7.6E^−10^	13.818	5.472–34.896	2.8E^−8^
MiR-34a level	0.030	0.001–1.587	0.083	0.030	0.000–1.963	0.100
WNT1 expression	6.497	1.992–21.966	0.003	9.015	2.091–38.868	0.003
Multivariate analyses						
FIGO stage	4.413	1.639–11.883	0.003	4.807	1.711–13.503	0.003
Stromal invasion	2.625	0.611–11.280	0.195	2.599	0.597–11.321	0.203
Parametrial extension	2.114	0.666–6.711	0.204	1.789	0.600–5.332	0.297
Endometrial extension	0.558	0.109–2.865	0.485	–	–	–
LVSI	1.228	0.364–4.136	0.740	1.129	0.322–3.953	0.850
Surgical margin	0.814	0.246–2.697	0.736	0.445	0.121–1.631	0.222
LNM	6.998	2.387–20.518	3.9E^−4^	5.517	1.766–17.235	0.003
WNT1 expression	3.527	1.015–12.251	0.047	4.394	0.985–19.601	0.052

FIGO, International Federation of Gynecology and Obstetrics; LVSI, lymph vascular space invasion; LNM, lymph node metastasis; HR, hazard ratio; 95% CI, 95% confidence interval; HR-HPV, high-risk human papillomavirus

## Discussion

In the past decades, cervical cancer has been studied extensively. WNT/β-catenin pathway activation was found to be present in >70% of the cases and to play an important role in their occurrence and progression. Specifically, WNT inhibitory factor 1, a secreted WNT antagonist, was found to be downregulated in human cervical cancer tissues, and to be able to inhibit cervical cancer cell proliferation and invasion in vitro and in vivo through inhibition of the WNT/β-catenin pathway [19]. WNT1 is a key member of the WNT family. The WNT1 protein was initially identified as a virus-induced proto-oncogene in mouse mammary tumors. Subsequent accumulating evidence revealed its important role in a variety of malignancies, including the cervical adenocarcinoma cell line Hela [20]. As yet, however, its aberrant expression has not been reported in cervical SCC tissues. We found that the expression of WNT1 was significantly upregulated in cervical SCC tissues relative to normal cervical tissues and correlated with a poor prognosis. In addition, we found that decreased WNT1 expression in HPV-16 positive cervical SCC cells inhibited their proliferation, migration and invasion, blocked their cell cycle in the G1 phase and promoted their apoptosis. These data suggest that WNT1 may function as a potential oncogene in cervical SCC cells.

Epithelial mesenchymal transition (EMT) is a physiological process that operates in the context of embryogenesis, wound healing and cancer development, driving epithelial cells to a mesenchymal state [21]. With their loss of polarity and connection with the basement membrane via EMT, tumor cells acquire higher migration and invasion capacities. However, the molecular mechanism underlying EMT remains complex, and a series of signaling molecules regulating EMT has been identified, including TGF-β, ERK/p-ERK, MAPK/p-MAPK, AKT/p-AKT, Smad/p-Smad, Notch and WNT/β-catenin [22, 23]. In recent years, the WNT/β-catenin pathway has gradually gained attention in HR-HPV induced cervical cancer [24–26]. The classical cadherin family is comprised of various proteins, including E-cadherin, N-cadherin and P-cadherin, all of which are required for normal cell-cell adhesion. A number of studies has shown that deregulation and abnormal expression of classical cadherins may result in aberrant adherent junctions, eventually contributing to the initiation and progression of malignancies [27]. More importantly, cadherin switches (referred to as switches from E-cadherin to P-cadherin or E-cadherin to N-cadherin) have been regarded as key hallmarks of EMT [28] and to be involved in TGF-β signaling-induced EMT [29]. Previously, we found that aberrant E-cadherin expression led to increased migration and invasion of cervical SCC cells and could serve as a key biomarker for a poor prognosis of early-stage cervical SCC [7, 8]. Furthermore, we found that P-cadherin could affect the expression of E-cadherin and p120 catenin, thereby contributing to cervical cancer initiation and progression [30]. These results suggested an important role of the E-P cadherin switch in cervical SCC development. The regulatory mechanism underlying the interaction between the E-P cadherin switch and cervical SCC development remained, however, unidentified. Our current data revealed that a reduction in WNT1 expression resulted in E-cadherin upregulation and P-cadherin downregulation, as well as downregulation of total and nuclear β-catenin protein expression in Siha and Caski cells. In addition, we found that the expression of the WNT/β-catenin pathway downstream genes AXIN2, BMP4 and FGF9 was significantly decreased in WNT1-silenced Siha and Caski cells. LiCl is a well-defined activator of the WNT/β-catenin pathway, increasing total and nuclear β-catenin expression [31]. We found that aberrant E-cadherin and P-cadherin expression induced by WNT1 silencing could partially be reversed by LiCl treatment. Therefore, we conclude that WNT1 regulates the E-P cadherin switch and, subsequently, promotes cervical SCC cell growth and invasion, at least partly through WNT/β-catenin pathway activation.

MicroRNAs (miRNAs) constitute a class of small endogenous non-coding RNAs with a length of approximately 18–25 nucleotides that may affect target gene expression at both the transcriptional and post-transcriptional level [32, 33]. Cervical cancer, like many other cancers, has been found to exhibit aberrant expression of both oncogenic and tumor suppressive miRNAs. MiR-34a is one of the most informative miRNAs among these miRNAs. Its level in cervical lesions and cancer has been shown to serve as a predictive biomarker for distinguishing different HR-HPV infected cervical lesion types [34]. Furthermore, it has been reported to serve as a potential therapeutic target [35]. Several studies have provided evidence that miRNAs can regulate the WNT/β-catenin pathway by directly targeting WNT components [36]. However, exactly determining the association between miR-34a expression and the WNT/β-catenin pathway in cervical SCC is challenging. A recent study, using both bioinformatic target prediction and dual-luciferase reporter assays, has suggested that WNT1 may be a direct downstream target of miR-34a [15]. Here, a negative correlation between miR-34a and WNT1 expression in 131 cases of cervical SCC was noted, and miR-34a was found to be associated with adverse prognostic factors. Furthermore, we exogenously upregulated the expression of miR-34a in cervical cancer cell lines and found, both in vitro and in vivo, that the mRNA and protein levels of WNT1 were significantly decreased. Therefore, we conclude that WNT1 may serve as a target of miR34a in cervical SCC and can be regulated by this miRNA at the transcriptional level. We also examined the levels of total and nuclear β-catenin, and expression of the downstream genes AXIN2, BMP4 and FGF9 of the WNT/β-catenin pathway, and found that all of them decreased after transfection of a miR-34a mimic into Siha and Caski cells. More importantly, we observed increased E-cadherin and decreased P-cadherin expression levels in miR-34a overexpressing Siha and Caski cells, both in vitro and in vivo. Finally, we examined the proliferation, invasion and growth of cervical SCC cells and found that miR-34a overexpression decreased WNT1 expression and inhibited the proliferation, invasion and growth, and promoted apoptosis of these cells both in vitro and in vivo. Therefore, our results suggest that miR-34a can inactivate downstream factors of the WNT/β-catenin pathway through directly targeting WNT1, thereby altering cervical SCC cell growth, development and invasion by regulating the E-P cadherin switch.

High-risk (HR) HPV persistent infections have been shown to be key drivers of cervical cancer initiation and progression, but different HR-HPV types may have different oncogenic activities [37]. HPV-16/18 are most carcinogenic among the different HR-HPV types. Although we found no statistically significant association between WNT1 or miR-34a levels and HR-HPV infection status in 131 primary cervical SCC tissues, we did find that the HPV-16/18 positive cases exhibited a more aberrant expression of miR-34a and WNT1 than the other HR-HPV positive cases. Furthermore, we found that siRNA mediated HPV-16 E6/E7 silencing resulted in miR-34a upregulation and WNT1 downregulation in HPV-16 positive Siha and Caski cells. These results indicate that HPV-16 E6/E7 may be involved in the processes regulating miR-34a and WNT1 expression in cervical SCC cells. Future investigations aimed at resolving correlations between HPV-16 E6/E7 expression levels and miR-34a-mediated modulation of the WNT1/β-catenin and E-P cadherin switch pathways may be instrumental to resolve the molecular mechanisms underlying cervical SCC initiation and progression.

Patients with early-stage cervical SCC have a relatively better prognosis. The major prognostic factors affecting survival among patients with cervical SCC are stage, nodal status, tumor volume, depth of cervical stromal invasion and LVSI. Disease stage is the most important prognostic factor, followed by lymph node status. In order to improve the survival, the effect of several molecular alterations has been studied in early-stage cervical SCC. The most relevant alterations were found in E-cadherin [7], ROR2 [38], URG4 [39] and Ubiquitin-specific protease 22 [40]. Here, we found that WNT1 and miR-34a are associated with both DFS and OS in 131 patients with early-stage cervical SCC and, most importantly, both univariate and multivariate analyses confirmed that WNT1 serves as an independent prognostic biomarker of SCC, similar to FIGO staging and LNM. Hence, our results suggest that WNT1, as a target of miR-34a, may serve as a biomarker for a poor survival of early-stage cervical SCC patients.

## Conclusions

From our data, we found that WNT1 acts as a cervical SCC promoting factor regulating E-P cadherin switching through the WNT/β-catenin pathway, thereby promoting tumor cell growth and invasion. MiR-34a downregulation may activate downstream genes of the WNT/β-catenin pathway and enhance the E-P cadherin switch, thereby contributing to cervical SCC initiation and progression. HPV-16 E6/E7 may simultaneously regulate miR-34a and WNT1 expression in HPV-16 positive cervical SCC cells. Further studies are required to clarify the exact underlying mechanism and to design targeted miR-34a and WNT1-based therapies.

## Electronic supplementary material

ESM 1 (DOCX 85 kb)

ESM 2 (DOCX 36 kb)

ESM 3 (DOC 31 kb)

ESM 4 (DOCX 33 kb)

Supplementary Figure 1 The WNT1 level was higher as determined by Western blotting (**A**), while that of miR-34a was lower as determined by qRT-PCR (**B**) in HPV-16 positive Siha and Caski cells than those in HPV negative C33A cells. (**C**) The expression of N-cadherin was tested by Western blotting in Siha and Caski cells with and without siR-WNT1 or miR-34a mimics treatment. The protein level of N-cadherin was too low to be detected in these cells. Increased miR-34a levels using miR-34a mimics successfully suppressed the miRNA (**D**) and protein (**E**) expression of WNT1 in Siha and Caski cells. (PNG 529 kb)

High resolution image (TIF 40890 kb)
