# Supplementary material for: WNT1, a target of miR-34a, promotes cervical squamous cell carcinoma proliferation and invasion by induction of an E-P cadherin switch via the WNT/β-catenin pathway
Source: Cell Oncol (Dordr). 2020 Apr 16;43(3):489–503. doi: 10.1007/s13402-020-00506-8 (PMC7214512; doi:10.1007/s13402-020-00506-8)
Supplement: Supplementary file 1 — (DOCX 85 kb) [file 13402_2020_506_MOESM1_ESM.docx]

Supplementary Table 1 Sequences of Primers and siRNAs

| Primers and siRNAs | Sequence |
| --- | --- |
| siRNA targeting WNT1 | 5’-GGTTCCATCGAATCCTGCA-3’ |
| Promoter targeting siRNA HPV-16 E6/E7 forward | 5’-GUAACCGAAAUCGGUUGAACC-3’ |
| Promoter targeting siRNA HPV-16 E6/E7 reverse | 5’-CAUUGGCUUUAGCCAACUUGG-3’ |
| GAPDH forward | 5’-GACAGTCAGCCGCATCTTCT-3’ |
| GAPDH reverse | 5’-TTAAAAGCAGCCCTGGTGAC-3’ |
| HPV-16 E6 forward | 5’-CTGCAAGCAACAGTTACTGC-3’ |
| HPV-16 E6 reverse | 5’-GGCTTTTGACAGTTAATACACC-3’ |
| HPV-16 E7 forward | 5’-CATGGAGATACACCTACATTGC-3’ |
| HPV-16 E7 reverse | 5’-CACAACCGAAGCGTAGAGTC-3’ |
| WNT1 forward | 5’-CTGGAACTGTCCCACTGCTC-3’ |
| WNT1 reverse | 5’-GGATTCGATGGAACCTTCTG-3’ |
| AXIN2 forward | 5’-CAAGGGCCAGGTCACCAA-3’ |
| AXIN2 reverse | 5’-CCCCCAACCCATCTTCGT-3’ |
| BMP4 forward | 5’-GACTTCGAGGCGACACTTCTG-3’ |
| BMP4 reverse | 5’-GAATGACGGCACTCTTGCTA-3’ |
| FGF9 forward | 5’-CCCAACGGTACTATCCAGGGA-3’ |
| FGF9 reverse | 5’-AGGCCCACTGCTATACTGATAAA-3’ |
