# Supplementary material for: WNT1, a target of miR-34a, promotes cervical squamous cell carcinoma proliferation and invasion by induction of an E-P cadherin switch via the WNT/β-catenin pathway
Source: Cell Oncol (Dordr). 2020 Apr 16;43(3):489–503. doi: 10.1007/s13402-020-00506-8 (PMC7214512; doi:10.1007/s13402-020-00506-8)
Supplement: Supplementary file 2 — (DOCX 36 kb) [file 13402_2020_506_MOESM2_ESM.docx]

Supplementary Table 2 The information of the primary antibodies in the study

| **protein name** | **information** |
| --- | --- |
| WNT1 | Abcam (Ab63934) |
| E-cadherin | Santa Cruz (Sc-8426) |
| P-cadherin | Santa Cruz (Sc-74545) |
| N-cadherin | CST (13116S) |
| Nuclear beta catenin | Millipore (04-1070) |
| Beta catenin | Abcam (Ab16051) |
| GAPDH | Abcam (Ab9485) |
