# Supplementary material for: WNT1, a target of miR-34a, promotes cervical squamous cell carcinoma proliferation and invasion by induction of an E-P cadherin switch via the WNT/β-catenin pathway
Source: Cell Oncol (Dordr). 2020 Apr 16;43(3):489–503. doi: 10.1007/s13402-020-00506-8 (PMC7214512; doi:10.1007/s13402-020-00506-8)
Supplement: Supplementary file 3 — (DOC 31 kb) [file 13402_2020_506_MOESM3_ESM.doc]

| Characteristic | No. | WNT1, n (%) | | χ^2^ | *p*-value |
| --- | --- | --- | --- | --- | --- |
|  |  | Low | High |  |  |
| Normal cervix  SCC | 50  131 | 48 (26.5)  62 (34.3) | 2 (1.1)  69 (38.1) | 35.96 | 2.01E-9 |

Supplementary Table 3. Expression of WNT1 protein in normal cervix and SCC tissues

SCC, cervical squamous cell carcinoma.
