# Supplementary material for: WNT1, a target of miR-34a, promotes cervical squamous cell carcinoma proliferation and invasion by induction of an E-P cadherin switch via the WNT/β-catenin pathway
Source: Cell Oncol (Dordr). 2020 Apr 16;43(3):489–503. doi: 10.1007/s13402-020-00506-8 (PMC7214512; doi:10.1007/s13402-020-00506-8)
Supplement: Supplementary file 4 — (DOCX 33 kb) [file 13402_2020_506_MOESM4_ESM.docx]

Supplementary Table 4 The correlation of the expressions of miR-34a and WNT1 in 131 patients with cervical squamous cell carcinoma

|  | WNT1, n (%) | | χ^2^ | *p*-value |
| --- | --- | --- | --- | --- |
|  | Low | High |  |  |
| miR-34a, n (%) |  |  | 6.618 | 0.01 |
| Low | 40 (30.5) | 58 (44.3) |  |  |
| High | 22 (16.8) | 11 (8.4) |  |  |
