# Supplementary figures and images for: WNT1, a target of miR-34a, promotes cervical squamous cell carcinoma proliferation and invasion by induction of an E-P cadherin switch via the WNT/β-catenin pathway
Source: Cell Oncol (Dordr). 2020 Apr 16;43(3):489–503. doi: 10.1007/s13402-020-00506-8 (PMC7214512; doi:10.1007/s13402-020-00506-8)

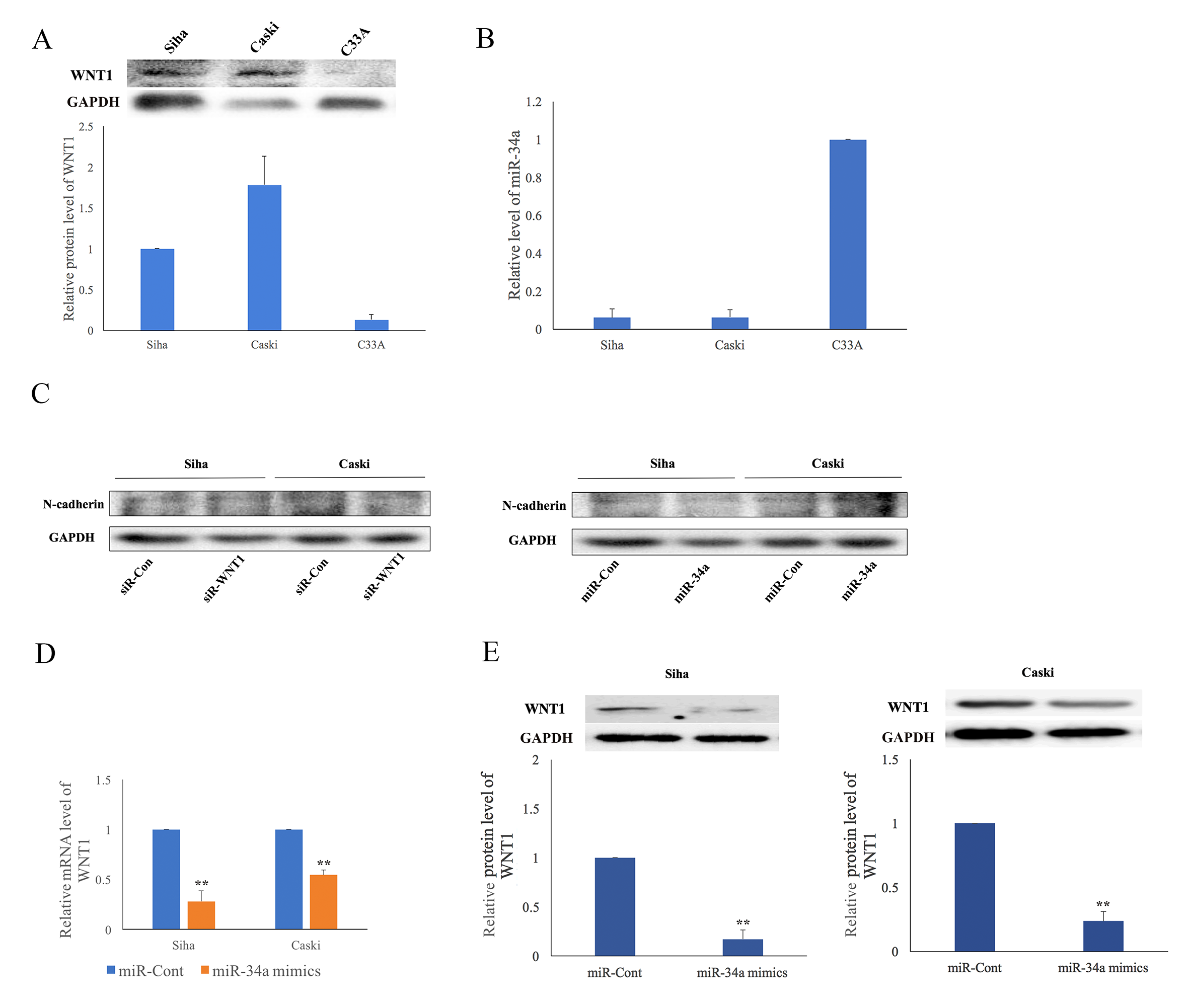

Supplement: Supplementary file 5 — The WNT1 level was higher as determined by Western blotting (A), while that of miR-34a was lower as determined by qRT-PCR (B) in HPV-16 positive Siha and Caski cells than those in HPV negative C33A cells. (C) The expression of N-cadherin was tested by Western blotting in Siha and Caski cells with and without siR-WNT1 or miR-34a mimics treatment. The protein level of N-cadherin was too low to be detected in these cells. Increased miR-34a levels using miR-34a mimics successfully suppressed the miRNA (D) and protein (E) expression of WNT1 in Siha and Caski cells. (PNG 529 kb) [file 13402_2020_506_Fig8_ESM.png]

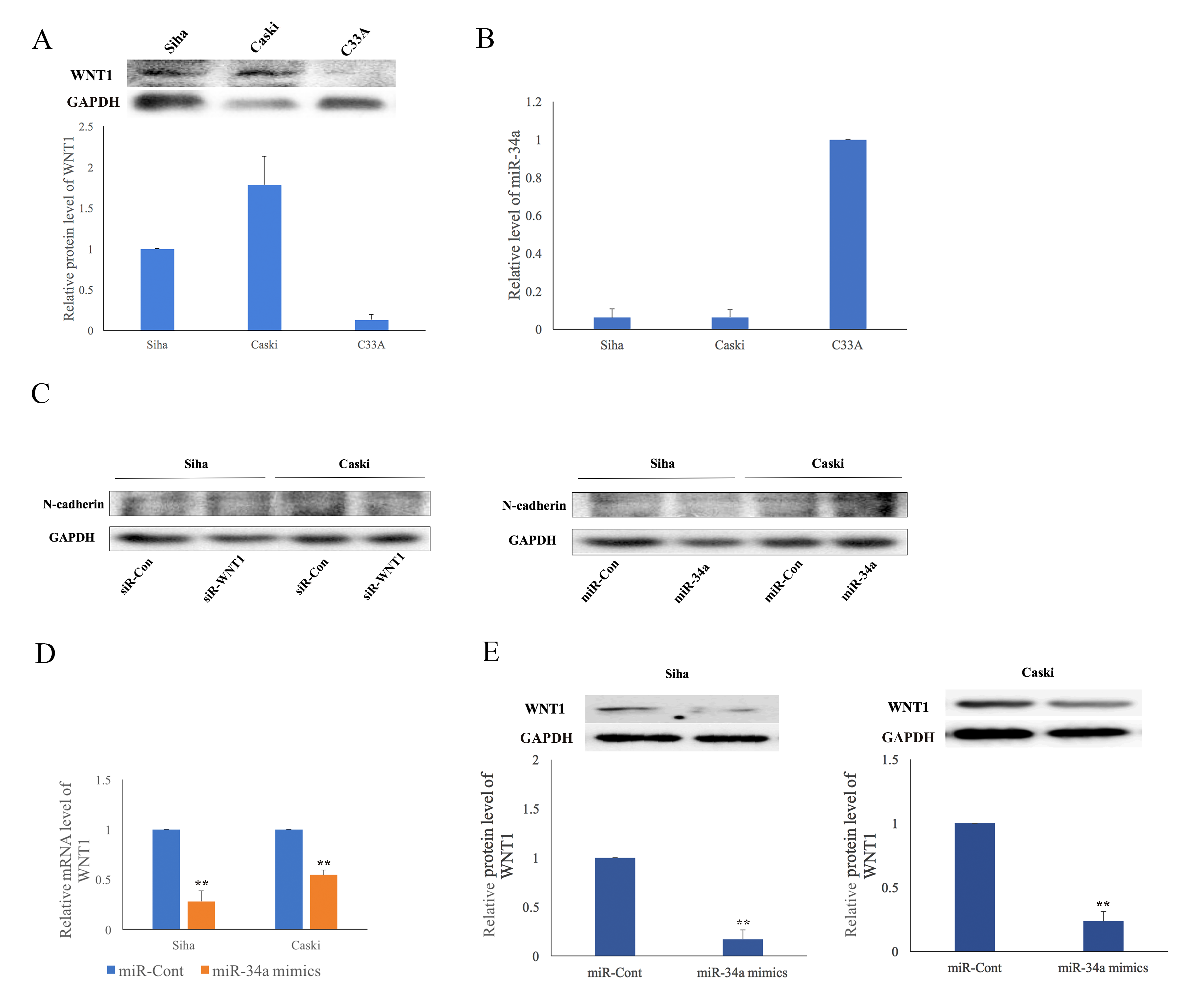

Supplement: Supplementary file 6 — High resolution image (TIF 40890 kb) [file 13402_2020_506_MOESM5_ESM.tif]
